# The impact of extramedullary and paraskeletal plasmacytomas on treatment outcomes in multiple myeloma treated with teclistamab: U.S. Myeloma Immunotherapy Consortium real-world experience

**DOI:** 10.1038/s41408-025-01414-6

**Published:** 2025-11-28

**Authors:** Aimaz Afrough, Danai Dima, Beatrice Razzo, Utkarsh Goel, Aishwarya Sannareddy, Oren Pasvolsky, Mariola A. Vazquez-Martinez, Christopher J. Ferreri, Rahul Banerjee, Jack Khouri, James A. Davis, Mahmoud R. Gaballa, Alex Lieberman-Cribbin, Masooma S. Rana, Kelley Julian, Faiz Anwer, Leyla Shune, Shaun DeJarnette, Ariel F. Grajales-Cruz, Evguenia Ouchveridze, Gabriel De Avila, Sandra P. Susanibar-Adaniya, Andrew J. Portuguese, Daniel Schrum, Erin Eberwein, Hitomi Hosoya, Lekha Mikkilineni, Gurbakhash Kaur, Joseph P. McGuirk, Adriana Rossi, Megan M. Herr, Omar Castaneda, Frederick L. Locke, Shahzad Raza, Yi Lin, Shebli Atrash, Douglas W. Sborov, Peter M. Voorhees, Shambavi Richard, Alfred L. Garfall, Surbhi Sidana, Krina K. Patel, Doris K. Hansen, Andrew J. Cowan, Larry D. Anderson, Hans C. Lee

**Affiliations:** 1 Hematologic Malignancies and Cellular Therapy Program, Simmons Comprehensive Cancer Center, UT Southwestern Medical Center, Dallas, TX USA; 2 Fred Hutchinson Cancer Center, Seattle, WA USA; 3 Abramson Cancer Center, University of Pennsylvania, Philadelphia, PA USA; 4 Thomas Jefferson University, Philadelphia, PA USA; 5 Cleveland Clinic, Taussig Cancer Center, Lerner College of Medicine, Cleveland, OH USA; 6 University of Texas MD Anderson Cancer Center, Houston, TX USA; 7 H. Lee Moffitt Cancer Center and Research Institute, Tampa, FL USA; 8 Atrium Health Levine Cancer Institute, Wake Forest University School of Medicine, Charlotte, NC USA; 9 The Medical University of South Carolina, Charleston, SC USA; 10 Icahn School of Medicine at Mount Sinai, New York, NY USA; 11 Stanford University, Palo Alto, CA USA; 12 Huntsman Cancer Institute, University of Utah, Salt Lake City, UT USA; 13 University of Kansas, Kansas City, KS USA; 14 Duke University, Durham, NC USA; 15 Roswell Park Comprehensive Cancer Center, Buffalo, NY USA; 16 Mayo Clinic, Rochester, MN USA

**Keywords:** Immunotherapy, Myeloma

## Abstract

Teclistamab, a bispecific antibody targeting B-cell maturation antigen (BCMA), is effective in relapsed or refractory multiple myeloma (RRMM), but its impact on patients with soft tissue plasmacytomas is unclear. We studied 385 RRMM patients treated with teclistamab at 13 U.S. centers through September 2023, with follow-up to April 2024. Soft tissue plasmacytomas were classified as true extramedullary disease (EMD; not contiguous with bone) or paraskeletal plasmacytomas (PSK; contiguous with bone). Patients with the simultaneous presence of both were classified as true-EMD, reflecting its adverse prognosis. Of those, 109 (28%) had true EMD, 33 (9%) had PSK, and 243 (63%) had no soft tissue plasmacytoma (No-STP). Median follow-up was 9.9 months. Overall response rates were 38% in true-EMD, 54.1% in PSK, and 62.4% in No-STP (*p* < 0.001). Median progression-free survival (PFS) was 1.4 months in true-EMD, 6.51 months in PSK, and 8.95 months in No-STP (*p* < 0.0001). Median overall survival (OS) was 9.54 months for true EMD, 13.1 months for PSK, and not reached in No-STP (*p* = 0.00012). In multivariable analysis, true-EMD was independently associated with inferior PFS and OS, while PSK showed numerically lower outcomes. These findings highlight the need for tailored strategies in patients with soft tissue plasmacytomas, particularly those with true-EMD.

## Introduction

Relapsed or refractory multiple myeloma, particularly in advanced stages, is associated with the development of aggressive disease features, including extramedullary disease (EMD). EMD develops within an immunosuppressive microenvironment characterized by poor tumor vascularization and stromal barriers that may impede effective T-cell function, posing significant treatment challenges.

True-EMD refers to soft tissue plasmacytomas arising from hematogenous dissemination without connection to bony involvement, commonly affecting sites such as the skin, muscle, liver, kidneys, lymph nodes, CNS, breast, pleura, and pericardium [1]. In contrast, paraskeletal disease (PSK) refers to bone-based plasmacytomas where tumor growth extends into soft tissue following cortical bone disruption. Historically, PSK and true-EMD were grouped together, but newer definitions distinguish true-EMD based on its hematogenous spread [1, 2].

The prevalence of true-EMD has increased in later treatment lines, while PSK remains stable from diagnosis [3]. However, that data is based on the era before the availability of T-cell therapies, and with improving survival, PFS, and shifting treatment landscapes, the true prevalence in the current era of T-cell therapy remains uncertain. Patients with true-EMD experience the shortest survival [4, 5], whereas PSK has historically had less severe, but still adverse, impact on survival [6]. These differences underscore the importance of accurate classification, particularly when evaluating T-cell redirection therapies.

Teclistamab, a bispecific antibody (BsAb) targeting CD3 on T cells and B-cell maturation antigen (BCMA) on myeloma cells, was approved in the United States (U.S.) in October 2022 for relapsed/refractory multiple myeloma (RRMM) for patients previously treated with four or more prior therapies, including an immunomodulatory agent (IMiD), a proteasome inhibitor (PI), and an anti-CD38 monoclonal antibody. In the pivotal MajesTEC-1 trial, EMD was defined as soft-tissue lesions not related to bone (true-EMD) [5]. Lesions arising from bone were excluded from EMD classification, in alignment with the previous definition [1]. Among the 165 patients treated with teclistamab, 17% had true-EMD, with a lower ORR of 35% compared to 63% in the overall population [5]. With a median follow-up of 30 months, updated results showed that among responders, the 24-month duration of response (DOR) was 50% in both the true-EMD group (10/28) and the overall recommended phase 2 dose (RP2D) cohort (104/165). Rates of death due to disease progression were higher in true-EMD (53.6%) compared to the overall RP2D population (33.9%) [4].

While MajesTEC-1 provided insights into true-EMD outcomes, it did not assess PSK separately or compare it to marrow-contained disease, who have no soft tissue plasmacytomas. Additionally, real-world patients often differ from clinical trial populations in terms of prior treatments, comorbidities, and response patterns. This study evaluated teclistamab’s real-world efficacy across distinct EMD classifications—true-EMD, PSK, and No-STP (without soft tissue plasmacytoma)—to address these gaps and provide a more comprehensive understanding of outcomes beyond controlled trial settings.

## Methods

This was a retrospective multicenter study, evaluating patients with RRMM at 13 medical centers in the U.S. Myeloma Immunotherapy Consortium, who received teclistamab by September 2023, with follow-up through April 30, 2024 (Supplementary Fig. 1—Consort diagram). Each center obtained independent Institutional Review Board approval and informed consent in accordance with institutional requirements.

Patients included in the study were classified into three groups based on center-reported data: (1) true-EMD, defined as soft tissue (visceral or non visceral) plasmacytomas non-contiguous with bone; (2) PSK, characterized by soft tissue extension from bone-based lesions; and (3) No-STP, patients without soft tissue plasmacytomas. Patients with both PSK and true-EMD simultaneously were categorized as true-EMD, due to its adverse prognosis. Patients were classified into three cytogenetic risk groups based on a modified version of the 2025 International Myeloma Society (IMS)/International Myeloma Working Group (IMWG) consensus criteria [7]: (1) standard risk, (2) confirmed high-risk, defined as t(4;14), t(14;16), or t(14;20) in combination with either gain/amp(1q) or del(1p), or concomitant gain/amp(1q) and del(1p) (including cases with concurrent del(17p)), and (3) isolated del(17p), categorized separately if not meeting criteria for confirmed high-risk, regardless of variant allele frequency or clonal fraction, given the absence of TP53 mutation data and incomplete FISH threshold information. In our study, patients reported with plasma cell leukemia (PCL) diagnosed between 2017 and 2022 were included based on chart documentation. During this period, the diagnostic criteria for PCL changed (from ≥2 × 10⁹/L circulating plasma cells before 2021 to ≥5% circulating plasma cells or ≥0.5 × 10⁹ cells/L of plasma cells in peripheral blood [8]; therefore, PCL classification was based on the documented diagnosis rather than uniform reapplication of updated definitions. Additionally, information regarding primary versus secondary PCL was not available in the dataset.

Response was assessed by treating investigators using the IMWG criteria [9]. Due to the retrospective nature of the study, not all IMWG criteria were strictly applied. Confirmatory testing for complete responses (CR) was not required, and therefore, some CRs may not fully meet IMWG criteria. Patients with hematologic response, but imaging progression, were classified as having progressive disease for the overall response rate (ORR) analysis. If imaging was unavailable during the best hematologic response, the response was determined based on available hematologic criteria. For patients with true-EMD or PSK, hematologic and radiographic responses were evaluated when available. Radiographic response was assessed based on available imaging modalities as reported by each center, recognizing variability in imaging timelines and methods across institutions. Imaging methods, including PET/CT, CT, or MRI, depending on institutional practice (Supplementary Table 1). Any increases in plasmacytomas during Cycle 1 were not considered disease progression to avoid misinterpreting tumor flares [10–12]. Patients who died before response assessment were classified as hematological non-responders.

### Statistical analysis

For the overall cohort, comparisons among groups of interest (true-EMD vs. PSK vs. No-STP) were conducted using chi-square tests or Fisher’s exact tests for categorical variables and Kruskal–Wallis rank-sum tests for continuous variables. Duration of response (DOR) was measured from the first documented response (PR or better) until disease progression. Patients without progression at the last follow-up or those who died without progression were censored. Progression-free survival (PFS) was defined as the time from initiation of teclistamab to progression or death, whichever occurred first. Patients who remained alive and free from progression were censored at their last follow-up. Overall survival (OS) was defined as the time from initiation of teclistamab until death. Survival distributions were estimated using the Kaplan–Meier method, and subgroups were compared using the log-rank test. Univariate Cox regression models were constructed, incorporating pre-specified covariates, including age at first teclistamab, Eastern Cooperative Oncology Group (ECOG) performance status at teclistamab, PCL at any time through the myeloma course, STP type at the time of teclistamab initiation, cytogenetic abnormalities per Fluorescence in situ hybridization (FISH) at any time, prior BCMA-directed therapy, triple-class, and penta-refractory status. Variables with a *p* value < 0.05 in the univariate analysis were included in the multivariable Cox proportional hazards regression model. All statistical analyses were conducted in R (version 4.3.1), and SPSS (V29.0). All statistical tests were two-sided, and a *p* value < 0.05 was considered statistically significant.

## Results

### Baseline characteristics

Of 385 patients treated with teclistamab, 109 (28%) had true-EMD, 33 (9%) had PSK, and 243 (63%) had No-STP at the time of teclistamab initiation. In the true-EMD cohort, 54% (*n* = 59) had visceral disease involvement, and 72% (*n* = 79) presented with more than one EMD lesion at teclistamab initiation. One third of patients with true-EMD had known concurrent PSK disease (*n* = 33, 30%), while the presence of PSK along with EMD is unknown for 38% (*n* = 41) of patients. In the PSK cohort, 45% (*n* = 15) had multiple disease sites.

The median age of the entire cohort was 68 years (range, 31.2–92), with 50% having received prior BCMA-directed therapy. The median time from MM diagnosis to teclistamab initiation was 5.8 years (interquartile range (IQR), 3.5–9.3 years).

Baseline characteristics are summarized in Table 1 and Supplementary Table 2, and were generally comparable across the three groups, except that patients with true-EMD and PSK were younger and had received more prior BCMA-directed therapies. There were no significant differences between groups regarding high-risk cytogenetics, penta-refractory disease, or median prior lines of therapy.

**Table 1:** Patient and disease characteristics based on STP type.

Characteristic, *N* = 385 (100%)	No.	True-EMD (*n* = 109, 28%)	PSK (*n* = 33, 9%)	No-STP (*n* = 243, 63%)	*p* value
Age in years, median, (range)	385	65.7 (31.2–88.7)	65.3 (39–84)	69.2 (37.1–92)	**0.007**
Female sex	385	49 (45%)	15 (45%)	118 (49%)	0.8
Race	385				
White	255	74 (68%)	19 (58%)	162 (67%)	0.5
Black	87	20 (18%)	10 (30%)	57 (23%)	
Other	43	15 (14%)	4 (12%)	24 (10%)	
Myeloma type	383				
IgA	77	24 (22%)	9 (28%)	44 (18%)	0.6
IgG	209	57 (53%)	17 (53%)	135 (56%)	
Light chain only	91	24 (22%)	6 (16%)	61 (25%)	
Others	6	3 (3%)	0	3 (1.2%)	
ECOG PS (*n* = 379)	376				
≥2	96	24 (23%)	11 (33%)	61 (26%)	0.4
ISS	385				0.7
Stage I	7	3 (2.7%)	0	4 (1.6%)	
Stage II	51	14 (13%)	2 (6%)	35 (14.4%)	
Stage III	37	12 (11%)	3 (9%)	22 (9%)	
Unknown/not reported	290	80 (73%)	28 (85%)	182 (75%)	
R2-ISS	385				0.3
Stage I	0	0	0	0	
Stage II	14	6 (5.5%)	1 (3%)	7 (3%)	
Stage III	58	20 (18%)	4 (12%)	34 (14%)	
Stage IV	20	3 (3%)	0	17 (7%)	
Unknown/not reported	293	80 (73%)	28 (85%)	185 (76%)	
Cytogenetic risk	362				
Standard risk	243	65 (64%)	22 (69%)	156 (68%)	0.29
Confirmed High-risk	65	24 (23.5%)	7 (22%)	34 (15%)	
Isolated 17p	54	13 (13%)	3 (9%)	38 (17%)	
Plasma cell leukemia (any time)	385	5 (5.0%)	1 (3%)	6 (2.5%)	0.5
Triple-class refractory	385	95 (87%)	27 (82%)	200 (82%)	0.5
Penta-refractory	385	44 (40%)	11 (33%)	89 (37%)	0.7
Number of prior lines, median (range)	375	6.5 (3–16)	6 (2–18)	6 (2–17)	0.19
Prior BCMA-directed therapy	385	68 (62%)	17 (51.5%)	108 (44%)	**0.008**

Not all percentages add up to 100% because of rounding. Triple-class refractory: defined as refractory to ≥1 immunomodulatory drug, ≥1 proteasome inhibitor, and ≥1 anti-CD38 monoclonal antibody. Penta-refractory: defined as refractory to ≥2 immunomodulatory drugs, ≥2 proteasome inhibitors, and ≥1 anti-CD38 monoclonal antibody. Cytogenetic risk: patients were classified into three cytogenetic risk groups based on a modified version of the 2025 IMS/IMWG consensus criteria: (1) standard risk, (2) confirmed high-risk, defined as t(4;14), t(14;16), or t(14;20) in combination with either gain/amp(1q) or del(1p), or concomitant gain/amp(1q) and del(1p) (including cases with concurrent del(17p)), and (3) isolated del(17p), categorized separately if not meeting criteria for confirmed high-risk, regardless of variant allele frequency or clonal fraction, given the absence of TP53 mutation data and incomplete FISH threshold information.

*STP* soft tissue plasmacytoma, *True-EMD* soft tissue plasmacytomas non-contiguous with bone, *PSK* paraskeletal, bone-based soft tissue, *BCMA* B-cell maturation antigen, *ECOG PS* Eastern Cooperative Oncology Group Performance Status, *R2-ISS* 2nd revision of ISS.

### Safety

Adverse effects are summarized in Table 2. There were no significant differences observed in the incidence and severity of cytokine release syndrome (CRS) between true-EMD, PSK, and No-STP (any grade: 52% vs. 54.5% vs. 59%, *p* = 0.3; grade ≥2: 8% vs. 6% vs. 12%, *p* = 0.4). There was no difference in median onset and duration of CRS across groups.

**Table 2:** Safety with standard of care teclistamab according to STP type.

Adverse event	No.	True-EMD (*n* = 109, 28%)	PSK (*n* = 33, 9%)	No-STP (*n* = 243, 63%)	*p* value
CRS	385				
Any grade CRS	219	57 (52%)	18 (54.5%)	144 (59%)	0.3
≥Grade 2 CRS	41	9 (8%)	2 (6%)	30 (12%)	0.4
Median time to start of CRS, days	219	3 (1–8)	3 (1–8)	2 (0–15)	0.079
Median duration of CRS, days^a^	219	0 (0–11)	0 (0–3)	0 (0–11)	0.2
ICANS	385				
Any grade ICANS	53	17 (16%)	5 (15%)	31 (13%)	0.7
≥Grade 2 ICANS	28	10 (9%)	5 (15%)	13 (5%)	**0.045**
Median time to start of ICANS, days	53	5 (0–25)	4 (1–21)	3 (0–14)	0.2
Cause of Tec discontinuation	278				**0.003**
MM or PD related	188	76 (82%)	15 (65%)	97 (60%)	
Infection	36	9 (10%)	1 (4%)	26 (16%)	
Others	54	8 (10%)	7 (30%)	39 (24%)	
Cause of death	137				
PD related	101	45/109 (41%)	8/33 (24%)	48/243 (20%)	0.14
NRM related	36	10/109 (9%)	2/33 (6%)	24/243 (10%)	

*CRS* cytokine release syndrome, *ICANS* immune effector cell-associated neurotoxicity syndrome, *NRM* non-relapse mortality.

^a^In CRS, a median time of 0 indicates that it lasted less than 1 day.

Values with *p*<0.05 are shown in bold.

There were no significant differences observed in the incidence of immune effector cell-associated neurotoxicity syndrome (ICANS) between true-EMD, PSK, and No-STP (any grade: 16% vs. 15% vs. 13%, *p* = 0.7), however, patients in the PSK group had a higher rate of ICANS grade ≥2 at 15%, compared to 9% in the true-EMD group and 5% in the No-STP group (*p* = 0.045). The median time to onset of ICANS was comparable across groups (*p* = 0.2).

There were no differences between groups regarding the use of tocilizumab, corticosteroids, or anakinra for either CRS or ICANS, except for a higher use of corticosteroid in the PSK group, which attributed to a greater incidence of high-grade ICANS. The primary cause of treatment discontinuation across all groups was disease progression, with a significantly higher incidence in the true-EMD group (82%) compared to 65% in the PSK group and 60% in the No-STP group (*p* = 0.003).

### Response rates

**Fig. 1:**
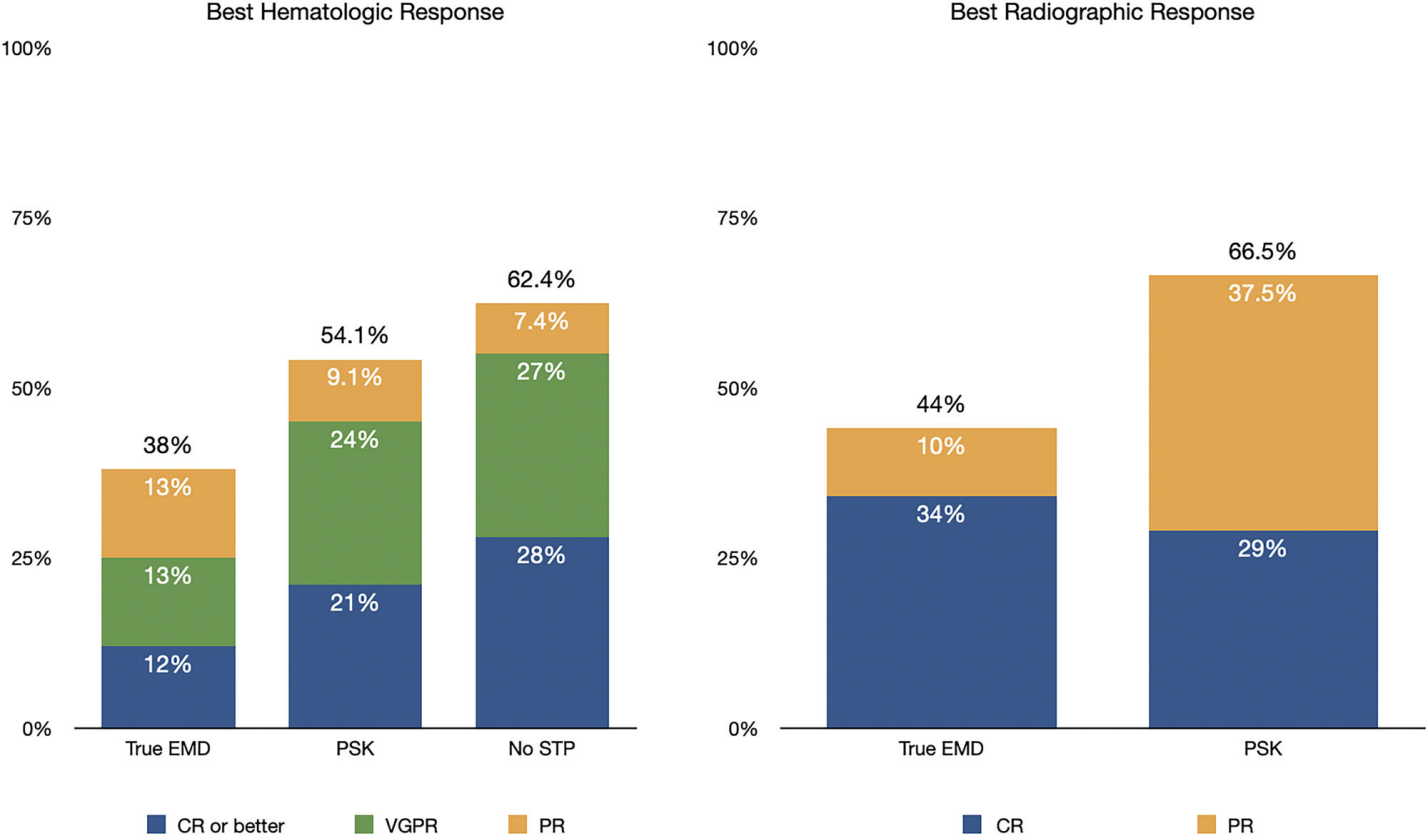
Response with teclistamab according to STP type. STP soft tissue plasmacytoma, CR complete response, VGPR very good partial response, PR partial response.

The hematologic and radiographic response rates for each STP group are presented in Fig. 1. The hematologic overall response rate (ORR; partial response [PR] or better) was 38% in the true-EMD group, 54.1% in the PSK group, and 62.4% in the No-STP group (*p* < 0.001). The rates of complete response (CR) or better were 12%, 21%, and 28% in the true-EMD, PSK, and No-STP groups, respectively (*p* = 0.006). Pairwise comparisons showed no significant difference in ORR between the No-STP and PSK groups (OR = 0.69, 95% CI: 0.3–1.4, *p* = 0.33), while patients with true-EMD had significantly lower odds of response compared with those without STP (OR = 0.37, 95% CI: 0.23–0.59, *p* < 0.001), corresponding to an approximately 63% decrease in odds of response. The comparison between true-EMD and PSK trended toward lower odds but was not statistically significant (OR = 0.53, 95% CI: 0.24–1.16, *p* = 0.11). Similar patterns were observed for CR (data not shown).

Radiographic data were available for 79% (112/142) of patients with soft tissue plasmacytoma (true-EMD or PSK) at baseline. Among patients who did not experience early death (<1 month) or progression within the first treatment cycle, radiographic follow-up was available in 87% (92/106).

Radiographic response was evaluable in 62% (*n* = 68) of the true-EMD group and 73% (*n* = 24) of the PSK group. Among the true-EMD group, 56% (38/68) had progression of disease (PD), or stable disease (SD) as best response, 10% (7/68) achieved PR, and 34% (23/68) achieved CR, with ORR of 44%. In the PSK group, 33% (8/24) had best response of PD, or SD, 37.5% (9/24) achieved PR, and 29% (7/24) achieved CR, with ORR of 66.5% (*p* = 0.009). Among the remaining 50 true-EMD or PSK patients, radiographic response was not evaluable if they died or progressed rapidly within the first month of treatment or unable to complete the first cycle of treatment for other reasons (38/50; 76%), lacked available imaging (12/50; 24%) (Supplementary Table 3).

The DOR was evaluable in 80% (169/210) of responders. The median DOR was 8.06 months (95% CI: 6.18–NR) for the true-EMD, 8.49 months (95% CI: 6.45–NR) for the PSK, and 12.86 months (95% CI: 11.05–NR) for the No-STP group (*p* = 0.011) (Supplementary Fig. 2). Pairwise comparisons showed a significantly shorter DOR in true-EMD compared to No-STP (*p* = 0.008), while differences between true-EMD and PSK (*p* = 0.6) and between PSK and No-STP (*p* = 0.1) were not statistically significant.

### Survival outcomes

**Fig. 2:**
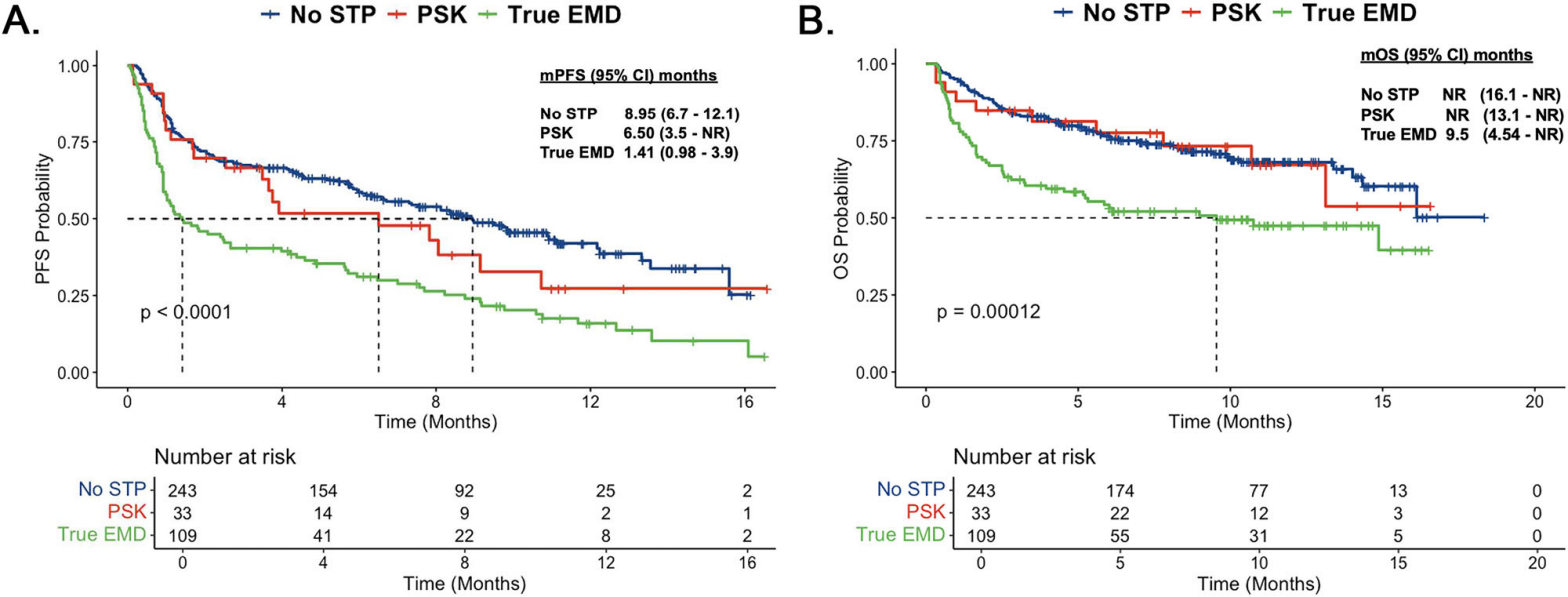
Survival outcomes according to STP. Kaplan–Meier estimates of PFS (**A**) and OS (**B**) in patients with RRMM who received teclistamab. Total of 137 events (progressions *n* = 101; deaths from any other causes *n* = 36); The median PFS was 1.4 months (95% CI: 0.98–3.98) for the true-EMD, 6.51 months (95% CI: 3.48–NR) for the PSK, and 8.95 months (95% CI: 6.7–12.1) for the No-STP group (*p* < 0.0001). The median OS was 9.54 months (95% CI: 4.54–NR) for the true-EMD, NR (95% CI: 13.12–NR) for the PSK, and NR for the No-STP group (95% CI: 16.1–NR) (*p* = 0.00012).

The median follow-up time from teclistamab initiation was 9.9 months (95% CI: 9.5–10.6 months). The median PFS for the entire cohort was 6.1 months (95% CI: 4.6–8.1); the median PFS was 1.4 months (95% CI: 0.98–3.98) for the true-EMD, 6.51 months (95% CI: 3.48–NR) for the PSK, and 8.95 months (95% CI: 6.7–12.1) for the No-STP group (*p* < 0.0001) (Fig. 2A). Pairwise comparisons showed no significant difference between No-STP and PSK groups (*p* = 0.28), while both true-EMD vs. No-STP (*p* = 0.000000014) and true-EMD vs. PSK (*p* = 0.041) were statistically significant.

The median OS for the entire cohort was 16.1 months (95% CI: 14.3–NR). By group, the median OS was 9.54 months (95% CI: 4.54–NR) for the true-EMD, NR (95% CI: 13.12–NR) for the PSK, and NR for the No-STP group (95% CI: 16.1–NR) (*p* = 0.00012) (Fig. 2B). Pairwise comparisons showed no significant difference between No-STP and PSK groups (*p* = 0.9). There was a trend toward worse OS between true-EMD compared with PSK (*p* = 0.074), while true-EMD had significantly worse OS compared with No-STP (*p* = 0.000084).

Across the cohort, 137 deaths were recorded, with 74% (101/137) primarily attributed to disease progression. Among those who died, progression accounted for 82% (45/55) in the true-EMD group, 80% (8/10) in the PSK group, and 67% (48/72) in the No-STP group. The cumulative incidence of non-relapse mortality (NRM) was 26% (36/137), with infection being the leading cause (47%,17/36). The distribution of NRM was similar across the groups, occurring in 9% (10/109) in the true-EMD, 6% (2/33) in the PSK, and 10% (24/243) in the No-STP group (Table 2).

### Factors predicting survival

We conducted a univariate analysis to assess the impact of various factors on PFS and OS. Variables with a *p* value < 0.05 in the univariate analysis were incorporated into the multivariable model. The results are summarized in Table 3.

**Table 3:** Univariate and multivariate analysis of PFS and OS for the entire cohort.

Variable	No.	PFS				OS			
		Univariable		Multivariable		Univariable	*p* value	Multivariable	*p* value
		HR (95% CI)	*p* value	HR (95% CI)	*p* value	HR (95% CI)		HR (95% CI)	
Age at teclistamab	385	0.98 (0.93–0.99)	**0.002**	0.98 (0.97–0.99)	**0.015**	0.98 (0.96–1.001)	0.059		
ECOG PS	376								
0–1	280	Ref		Ref		Ref		Ref	
≥2	96	1.86 (0.41–2.45)	**<0.001**	2.05 (1.53–2.75)	**<0.001**	2.67 (1.89–3.79)	**<0.001**	3.02 (2.1–4.3)	**<0.001**
Cytogenetic^a^	362								
Standard-risk	243	Ref		Ref		Ref		Ref	
Confirmed High-risk	65	1.7 (1.3–2.4)	**<0.001**	1.5 (1.08–2.08)	**0.016**	1.35 (0.87–2.08)	0.17	1.07 (0.70–1.73)	0.68
Isolated 17p	54	1.35 (0.93–1.9)	0.109	1.23 (0.84–1.82)	0.27	**1.62 (1.03–2.55)**	**0.034**	1.48 (0.92–2.35)	0.09
Triple-class refractory	385								
No	63	Ref				Ref			
Yes	322	1.44 (0.98–2.1)	0.058			1.54 (0.91–2.60)	0.1		
Penta refractory	385								
No	241	Ref				Ref		Ref	
Yes	144	1.2 (0.95–1.5)	0.11			1.40 (1.003–1.96)	**0.048**	1.50 (1.05–2.13)	**0.024**
Prior BCMA-directed therapy	385								
No	192	Ref		Ref		Ref			
Yes	193	2.3 (1.22–4.3)	**0.02**	1.2 (0.93–1.62)	0.14	1.27 (0.90–1.78)	0.158		
Prior line of therapy	385	1.02 (0.98–1.07)	0.2			1.003 (0.94–1.06)	0.9		
PCL at any time	385								
No	373	Ref		Ref		Ref		Ref	
Yes	12	2.88 (1.15–7.20)	**0.02**	1.9 (0.99–3.64)	0.052	2.96 (1.44–6.05)	**0.003**	2.68 (1.29–5.59)	**0.008**
STP type	385								
No-STP	243	Ref		Ref		Ref		Ref	
PSK	33	1.2 (0.78–2.02)	0.32	1.12 (0.68–1.83)	0.6	1.04 (0.54–2.02)	0.8	1.17 (0.6–2.3)	0.63
True-EMD	109	2.2 (1.67–2.8)	**<0.001**	1.93 (1.44–2.60)	**<0.001**	2.07 (1.46–2.95)	**<0.001**	2.40 (1.65–3.50)	**<0.001**

Triple-class refractory: defined as refractory to ≥1 immunomodulatory drug, ≥1 proteasome inhibitor, and ≥1 anti-CD38 monoclonal antibody. Penta-refractory: defined as refractory to ≥2 immunomodulatory drugs, ≥2 proteasome inhibitors, and ≥1 anti-CD38 monoclonal antibody.

*ECOG PS* Eastern Cooperative Oncology Group Performance Status, *PCL* plasma cell leukemia, *HR* hazard ratio, *CI* confidence interval.

^a^Patients were classified into three cytogenetic risk groups based on a modified version of the 2025 IMS/IMWG consensus criteria: (1) standard risk, (2) confirmed high-risk, defined as t(4;14), t(14;16), or t(14;20) in combination with either gain/amp(1q) or del(1p), or concomitant gain/amp(1q) and del(1p) (including cases with concurrent del(17p)), and (3) isolated del(17p), categorized separately if not meeting criteria for confirmed high-risk, regardless of variant allele frequency or clonal fraction, given the absence of TP53 mutation data and incomplete FISH threshold information.

Values with *p*<0.05 are shown in bold.

In the multivariable analysis of the entire cohort, true-EMD at the time of teclistamab (*p* < 0.001; HR = 1.93; 95% CI, 1.44–2.60) was independently associated with inferior PFS. Additionally, younger age, poor performance status at the time of teclistamab and confirmed high-risk cytogenetic were associated with worse PFS.

For OS, the multivariable analysis found true-EMD at the time of teclistamab (*p* < 0.001; HR = 2.40; 95% CI, 1.65–3.50) as an association with worse outcome. Additionally, penta-refractory disease, PCL at any time, and poor performance status at the time of teclistamab were associated with inferior OS. Consistent results for both PFS and OS were observed after excluding patients who had PCL at any time point (*N* = 12) from the analysis (Supplementary Table 4).

Within the true-EMD cohort only, multivariable analysis revealed that younger age was associated with worse PFS. Similarly, younger age, in addition to PCL at any time point, and poor performance status, were associated with inferior OS (Supplementary Table 5).

## Discussion

This multicenter retrospective study represents the first analysis of the impact of soft tissue plasmacytomas (true-EMD and PSK) on outcomes in a large cohort of RRMM patients treated with teclistamab. Our findings highlight that true-EMD is independently associated with inferior PFS and OS, providing valuable insights into the management of these complex cases.

True-EMD remains a strong predictor of poor prognosis even in the era of immune effector cell therapies such as BsAbs and chimeric antigen receptor T-cell (CAR-T) therapy. In the phase II KarMMa trial of idecabtagene vicleucel (ide-cel), 39% of patients had soft tissue plasmacytoma (true-EMD and PSK), with an ORR of about 70%, comparable to the 73% response rate in the overall population [13]. However, data on DOR and PFS specifically for patients with STP were not reported. Similarly, CARTITUDE-1, which evaluated ciltacabtagene autoleucel (cilta-cel), included 20% of patients with soft tissue plasmacytomas (13% true-EMD, 6% PSK), reporting a 100% ORR but shorter median DOR (12.9 months) and PFS (13.8 months) compared to the overall cohort (not reached). At 27 months, PFS and OS rates were lower in the STP subgroup (47.4% vs. 54.9% and 52.1% vs. 70.4%, respectively) [14]. A retrospective real-world study of cilta-cel beyond the 4th line found that true-EMD was independently associated with poorer outcomes. Patients with true-EMD (26% of the cohort) had significantly shorter median PFS (9.1 vs. 12.9 months in those without, *p* < 0.001). True-EMD was associated with inferior PFS (HR: 1.96, *p* = 0.009) and OS (HR: 1.88, *p* = 0.04) in multivariable analysis [15]. Other studies also confirmed worse outcomes for true-EMD with significantly shorter PFS and OS with BCMA CAR-T therapy [16–18]. Similarly, the MyCARe model, developed to predict early relapse after BCMA CAR-T therapy, identified the presence of true-EMD or PCL as a significant risk factor for early progression (HR: 1.92, *p* < 0.001) [19].

T cell engager anti-BCMA BsAb therapies have also shown poorer outcomes in soft tissue plasmacytomas. Understanding the factors driving this resistance is crucial for optimizing treatment strategies. The MajesTEC-1 trial, which led to approval of teclistamab, defined EMD as true-EMD and included 17% patients with EMD. EMD patients had a lower ORR of 35%, compared to 63% in the overall population; however, among responders, the 24-month DOR was comparable (50% in both groups) [5]. Similarly, the MagnetisMM-3 trial, which led to elranatamab’s approval, defined EMD more broadly (including both true-EMD and PSK), and reported an ORR of 38.5% in patients with soft tissue plasmacytomas, compared to 71.4% in those without. Despite lower initial responses, the 15-month DOR was similar (77.9% vs. 70.6%) [20]. This may suggest that while soft tissue plasmacytomas are less responsive initially, once a response is achieved, durability is maintained. However, limited data exist regarding the differential outcomes of PSK compared to true-EMD, with PSK often being inconsistently categorized in clinical trial datasets.

Our retrospective study confirms the negative impact of true-EMD on survival outcomes [16, 17]. By distinguishing true-EMD from PSK, our study emphasizes that only true-EMD carries an adverse prognostic significance, thereby refining the understanding of extramedullary disease in this setting. Although PSK did not emerge as an independent predictor of outcome, patients with PSK experienced numerically lower response rate, shorter DOR, and survival compared to those without STP. These findings suggest that PSK remains clinically relevant, even if not a standalone prognostic factor. Unlike prior studies that reported similar DOR among responders with and without soft tissue plasmacytomas, our findings indicate a shorter DOR in true-EMD patients. However, this should be interpreted with caution, as our timing of baseline first response for calculation of DOR was primarily based on hematologic response criteria, which were more readily available in the majority of cases. This could have influenced DOR estimates, particularly in the absence of standardized imaging at response confirmation. Additionally, the retrospective nature of our study limits direct comparison with prospective trials that have reported durable responses among soft tissue plasmacytoma responders. Our clinical findings align with existing research on the genomic complexity of soft tissue plasmacytoma, offering a clearer understanding of the progression and its impact on clinical outcomes. Genomic studies show that PSK and true-EMD have increased complexity compared to bone marrow-based myeloma, with true-EMD displaying the highest genomic complexity [21]. Transcriptomic analysis of true-EMD samples identified the co-occurrence of 1q21 gain/amplification and MAPK pathway mutations in 79% (11/14) of cases [22]. The CoMMpass dataset confirmed this correlation, showing that only KRAS mutations combined with 1q21 gain/amplification, rather than either alone at diagnosis, elevated the risk of developing soft tissue plasmacytomas (HR = 2.4, *p* = 0.011) [22]. Additionally, NRAS, KRAS, BRAF, and TP53 mutations were identified in true-EMD cases, highlighting the potential for targeting the RAS-MAPK pathway as a therapeutic strategy [23]. While our study lacks access to pathway mutation data, we specifically evaluated 1q gain/amplification and found no significant difference between our compared groups (data not shown), suggesting that additional genomic drivers may be at play in our cohort.

In our study, prior BCMA-directed therapy was linked to lower PFS in univariable analysis but lost significance in multivariable analysis, suggesting other risk factors may diminish its impact. This aligns with real-world cilta-cel data (*p* = 0.08) showing a similar trend [15]. However, the strong association of true-EMD with inferior PFS and OS underscores its critical role in treatment decisions. Importantly, although patients with true-EMD were younger and had received more prior BCMA-directed therapies, the inferior outcomes observed were unlikely to be solely attributable to prior treatment history or underlying disease burden. Rather, our findings suggest that teclistamab monotherapy may be insufficient to fully overcome the aggressive biology associated with true-EMD. The strong association of true-EMD with inferior PFS and OS underscores its critical role in treatment decisions. Similar trends have been observed in real-world data [24], which reported that the presence of true-EMD/PCL is an independent adverse prognostic factor in patients treated with BCMA-directed therapies, associated with worse PFS and OS. Notably, although outcomes varied by drug class—with CAR-T therapy achieving the best survival, followed by T-cell engagers (TCEs), and Antibody-drug-conjugates (ADCs) performing the least favorably—the negative impact of true-EMD/PCL persisted across all treatment modalities. Importantly, our sensitivity analysis demonstrated that true-EMD remains independently associated with inferior PFS and OS even when patients with PCL were excluded (Supplementary Table 4), highlighting the prognostic impact of EMD regardless of PCL status.

Several strategies are being investigated to enhance BsAb efficacy across multiple patient subgroups, including those with high-risk features such as true-EMD and PSK. First, debulking strategies have been explored to enhance immunotherapy efficacy. A case report described a patient requiring high-dose steroids and radiation (XRT) for spinal cord compression post-BCMA CAR-T, where CRS-like symptoms and inflammatory spikes coincided with >30% increased T-cell receptor (TCR) diversity, suggesting radiation-induced synergy [25]. Similarly, chemotherapy-based debulking has been investigated. Preclinical studies showed that prior chemotherapy impairs T-cell function by damaging mitochondrial reserves, reducing proliferation and persistence [26, 27]. Clinically, our retrospective analysis of bridging therapy before ide-cel found worse PFS in patients receiving intensified/infusional alkylators (primarily cyclophosphamide [Cy]) [28]. However, in contrast to CAR-T, in vivo data suggest Cy enhances BCMA BsAb therapy by reducing the exhausted T cells and regulatory T cells and preserving a functional naïve/central memory T-cell pool [29]. While a small retrospective study supported a Cy combination debulking strategy prior to BsAb [30], further validation is needed. Our study did not include data on prior alkylator use before teclistamab, limiting direct comparisons. Additionally, 12% of true-EMD patients in our cohort received XRT during teclistamab therapy, with no significant difference in PFS between the XRT and non-XRT groups. However, given the small sample size, this finding should be interpreted with caution. Second, combination therapy may enhance efficacy. In the Phase 1b RedirecTT-1 trial, teclistamab combined with the anti-GPRC5D BsAb, talquetamab, showed promising results. In the RP2D cohort, the ORR was 61% in true-EMD patients (definition was restricted to bone-independent lesions ≥2 cm) versus 92% in those without. The estimated 18-month PFS rate was 53% for true-EMD and 70% in the overall RP2D cohort, supporting its potential in these high-risk patients [31]. Overall, while retrospective data and early-phase studies suggest that debulking or combination strategies may enhance bispecific antibody efficacy, prospective trials are needed to validate these approaches across different patient subgroups, including those with true-EMD and PSK.

Our study’s strengths include its large, diverse cohort treated with teclistamab across multiple institutions, enhancing generalizability. However, as a retrospective analysis, it faces limitations such as potential selection bias, missing data, and variability in investigator-led response assessments. First, CR definitions were based on investigator assessment without confirmatory bone marrow or imaging in most cases, so rates may not reflect strict IMWG-defined CR. Second, there was no uniform requirement for whole-body imaging (e.g., PET/CT, whole-body MRI, or CT) at baseline or during follow-up, and imaging was performed and reported at the discretion of treating investigators. As a result, while baseline imaging was documented in a subset of patients (including 72% with true-EMD, 100% with PSK, and 38% with No-STP), some patients may have been misclassified as not having EMD or PSK due to unreported or undocumented imaging. Third, the dosing frequency and schedule of teclistamab were not standardized and were left to investigator discretion, which may have affected response timing and outcomes. Furthermore, the absence of a standardized imaging protocol for plasmacytoma evaluation, with responses often determined by hematologic criteria when imaging was unavailable, may introduce discrepancies, particularly between serological and plasmacytoma responses [32].

Nevertheless, the multicenter real-world nature of the cohort offers valuable insights into teclistamab outcomes, including in this high-risk subgroup.

In conclusion, our data underscores the prognostic significance of true-EMD in patients treated with teclistamab. PSK was not an independent prognostic factor, but its potential impact on response and disease progression warrants further study. Future clinical trials should differentiate true-EMD from PSK to better characterize disease patterns. Strategies targeting true-EMD, including localized debulking or rational combination therapies, may help optimize outcomes, with careful consideration of PSK when relevant.

## Supplementary information

Supplemental Material

## Data Availability

Available from the corresponding author upon reasonable request.
