# Supplementary material for: The impact of extramedullary and paraskeletal plasmacytomas on treatment outcomes in multiple myeloma treated with teclistamab: U.S. Myeloma Immunotherapy Consortium real-world experience
Source: Blood Cancer J. 2025 Nov 28;16(1):12. doi: 10.1038/s41408-025-01414-6 (PMC12789570; doi:10.1038/s41408-025-01414-6)

Title: The Impact of Extramedullary and Paraskeletal Plasmacytomas on Treatment Outcomes in Multiple Myeloma treated with Teclistamab: U.S. Myeloma Immunotherapy Consortium Real-World Experience

Supplemental materials:

Supplemental Fig 1: Consort Diagram.

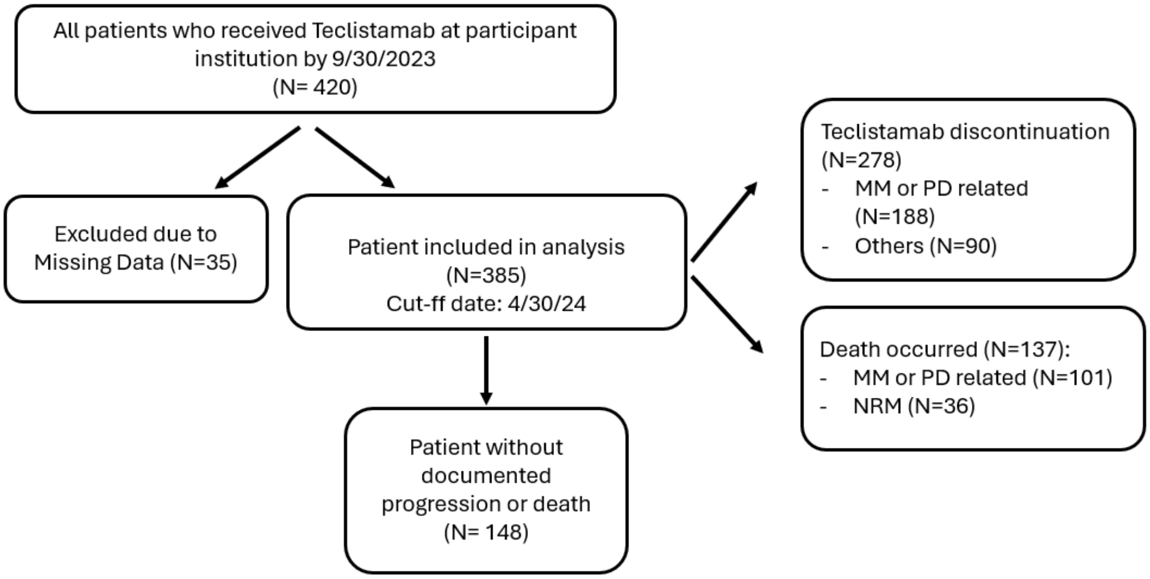

Supplemental table 1. Imaging Modality assessment in soft tissue plasmacytoma (STP).

Radiographic data were available for 79% (112/142) of patients with soft tissue plasmacytoma (true-EMD or PSK) at baseline. Among patients who did not experience early death (<1 month) or progression within the first treatment cycle, radiographic follow-up was available in 87% (92/106).

**Imaging modality**	**At baseline**<br>**N=112**	**Monitoring**<br>**N=92**
CT scan	18	19
MRI	20	10
PET/CT	65	37
Physical Exam (tumor palpable)	1	5
Unknow type	8	21

Supplemental Table 2: FISH abnormality based on STP type.

**FISH, N= 362 (94%)**	**True-EMD**<br>**(n=102)**	**PSK**<br>**(n=32)**	**No-STP**<br>**(n=228)**	**P value**
t(4;14)	13 (12%)	6 (19.5%)	25(10%)	0.3
t(14;16)	5 (5%)	2 (6%)	12 (5%)	0.9
t(14;20)	0	0	0	
t(11;14)	9 (9%)	4 (12.5%)	31 (13%)	0.5
Other IgH translocations	3 (3%)	0	11 (5%)	0.3
Del 17p	20 (20%)	6 (19%)	47 (20%)	0.9
1 q abnormalities	57 (56%)	15 (47%)	99 (42%)	0.06
1 p deletion	13 (13%)	2 (6%)	19 (8%)	0.3
Hyperdiploidy	21 (21%)	7 (22%)	60 (25.5%)	0.5
Other abnormality	36 (35%)	9 (28%)	78 (33%)	0.7
Negative FISH	38 (37%)	14 (44%)	99 (42%)	0.6

Supplemental Table 3: Hierarchical Table of Outcomes for true-EMD and PSK Groups

**Group**	**Early Death**	**PD<1 month or Short DOT**	**Imaging Available**	**Progression** per Imaging	**Progression** Pattern	**Soft Tissue Site (N)**
**True-EMD**	Yes (21, 19%)		—	—	—	—
	No (88, 81%)	Yes (10, 11%)	—	—	—	
			Yes (68,77%)	Yes (n=45, 66%)	Soft tissue only (16,35%)	Same site (17) New site (12) Both # (13) Unknown (3)
					Systemic* + EMD (28; 62%)	
					Unknown systemic status (1, 2%)	
				No/Unknown (n=23, 34%)	Systemic (5, 22%)	—
			No / Unknown (10, 11%)	NA	Systemic (5, 50%)	—
**PSK**	Yes (4, 12%)		—	—	—	—
	No (29, 89%)	Yes (3, 10%)				
			Yes (24, 83%)	Yes (11, 46%)	Soft tissue only (4,36%)	Same site (2) New site (4) Both # (4) Unknown (1)
					Systemic* + PSK (7, 64%)	
				No (13, 54%)	Systemic (2, 15%)	—
			No / Unknown (2, 7%)	NA	Systemic (2, 100%)	—

PD <1 months. If progression occurs within first cycle of treatment; DOT: duration of treatment

* “Systemic” progression was defined as disease progression based on biochemical markers (e.g., M-protein, light chains) or new/worsening lytic bone lesions, in the absence of progressive soft tissue plasmacytomas.

Both: progression on previous (known) site in addition to new site

Supplemental Table 4. Univariate and multivariate analysis of PFS and OS for patients <u>without</u> a history of PCL.

Variable	No.	PFS				OS			
		Univariable		Multivariable		Univariable		Multivariable	
		HR (95% CI)	P value	HR (95% CI)	P value	HR (95% CI)	P value	HR (95% CI)	P value
**Age at teclistamab**	373	0.98 (0.97-0.99)	**0.004**	0.98 (0.97-0.99)	**0.040**	0.98 (0.97-1.003)	0.099		
**ECOG PS**	364								
0-1	272	Ref		Ref		Ref		Ref	
≥2	92	1.84 (1.39-2.45)	**<0.001**	2.06 (1.49-2.72)	**<0.001**	2.65 (1.85-3.80)	**<0.001**	2.95 (2.05-4.26)	**<0.001**
**Cytogenetic***	350								
Standard-risk	237	Ref		Ref		Ref		Ref	
Confirmed High-risk	61	1.8 (1.3-2.5)	**<0.001**	1.63 (1.17-2.28)	**0.004**	1.40 (0.89-2.19)	0.139	1.07 (0.70-1.73)	0.68
Isolated 17p	52	1.32 (0.90-1.9)	0.144	1.18 (0.79-1.76)	0.41	1.57 (0.98-2.51)	0.058	1.48 (0.92-2.35)	0.09
**Triple refractory**	373								
No	61	Ref		Ref.		Ref			
Yes	312	1.50 (1.01-2.2)	**0.041**	1.40 (0.92-2.13)	0.106	1.56 (0.91-2.68)	0.1		
**Penta refractory**	373								
No	232	Ref				Ref		Ref	
Yes	141	1.2 (0.99-1.68)	0.060			1.47 (1.04-2.08)	**0.027**	1.55 (1.09-2.22)	**0.015**
**Prior BCMA-directed therapy**	373								
No	187	Ref		Ref		Ref			
Yes	186	1.3 (1.04-1.77)	**0.021**	1.2 (0.92-1.65)	0.15	1.27 (0.90-1.81)	0.165		
**Prior line of therapy**	363	1.02 (0.98-1.07)	0.19			1.01 (0.95-1.07)	0.73		
**STP type**	373								
No-STP	237	Ref		Ref		Ref		Ref	
PSK	32	1.3 (0.82-2.12)	0.246	1.19 (0.72-1.96)	0.484	1.08 (0.55-2.10)	0.55	1.06 (0.54-2.07)	0.85
True-EMD	104	2.1 (1.64-2.8)	**<0.001**	1.89 (1.39-2.56)	**<0.001**	1.98 (1.37-2.85)	**<0.001**	2.20 (1.51-3.22)	**<0.001**

Abbreviations and definitions: ECOG PS, Eastern Cooperative Oncology Group Performance Status; PCL, plasma cell leukemia; HR, Hazard ratio; CI, confidence interval; Triple-class refractory: defined as refractory to ≥1 immunomodulatory drug, ≥1 proteasome inhibitor, and ≥1 anti-CD38 monoclonal antibody. Penta-refractory: defined as refractory to ≥2 immunomodulatory drugs, ≥2 proteasome inhibitors, and ≥1 anti-CD38 monoclonal antibody

* Patients were classified into three cytogenetic risk groups based on a modified version of the 2025 IMS/IMWG consensus criteria: (1) **standard risk**, (2) **confirmed high-risk**, defined as t(4;14), t(14;16), or t(14;20) in combination with either gain/amp(1q) or del(1p), or concomitant gain/amp(1q) and del(1p) (including cases with concurrent del(17p)), and (3) **isolated del(17p),** categorized separately if not meeting criteria for confirmed high-risk, regardless of variant allele frequency or clonal fraction, given the absence of TP53 mutation data and incomplete FISH threshold information

Supplemental Table 5. Univariate and multivariate analysis of PFS and OS in true-EMD cohort.

Variable	N.	PFS				OS			
		Univariable		Multivariable		Univariable		Multivariable	
		HR (95% CI)	P value	HR (95% CI)	P value	HR (95% CI)	P value	HR (95% CI)	P value
**Age at Tec infusion**	109	0.97 (0.95-0.99)	**0.010**	0.97 (0.95-0.99)	**0.013**	0.97 (0.95-0.99)	**0.031**	0.97 (0.95-0.99)	**0.025**
**ECOG PS**	105								
0-1	81	Ref.				Ref.		Ref.	
≥2	24	1.55 (0.94-2.56)	0.08			2.4 (1.3-4.3)	**0.003**	2.3 (1.2-4.1)	**0.005**
**Cytogenetic risk***	102								
Standard risk	65	Ref.				Ref.			
Confirmed High-risk	24	1.31 (0.79-2.18)	0.29			1.55 (0.84-2.85)	0.15		
Isolated 17 p	13	1.4 (0.76-2.6)	0.25						
**Triple class refractory**	109								
No	14	Ref.				Ref.			
Yes	94	1.35 (0.69-2.63)	0.36			1.2 (0.5-2.8)	0.6		
**Penta refractory**	109								
No	65	Ref				Ref.			
Yes	44	1.09 (0.71-1.66)	0.68			1.05 (0.61-1.8)	0.83		
**Prior BCMA-directed therapy**	109								
No	41	Ref.				Ref.			
Yes	68	1.15 (0.74-1.79)	0.51			1.31 (0.74-2.31)	0.34		
**Prior line of therapy**	106	0.97 (0.89-1.06)	0.55			0.96 (0.86-1.07)	0.54		
**PCL at anytime**	109								
No	104	Ref.		Ref.		Ref.			
Yes	5	2.53 (1.02-6.2)	**0.045**	2.3 (0.95-5.9)	0.062	4.14 (1.6-10.5)	**0.003**	4.2 (1.6-11.0)	**0.003**
**Site of EMD**	109								
Non-Visceral	50	Ref.				Ref.			
Visceral	59	1.2 (0.83-1.88)	0.32			1.31 (0.7-2.2)	0.31		
**Number of EMD lesions**	109								
1	30	Ref.				Ref.			
2	6	1.3 (0.5-3.3)	0.49			2.04 (0.69-6.0)	0.19		
≥3	73	0.95 (0.5-1.5)	0.85			1.3 (0.68-2.8)	0.36		
**XRT to EMD site**	109								
No	96	Ref.				Ref.			
Yes	13	0.92 (0.47-1.80)	0.84			0.77 (0.30-1.94)	0.58		

Abbreviations and definitions: ECOG PS, Eastern Cooperative Oncology Group Performance Status; PCL, plasma cell leukemia; HR, Hazard ratio; CI, confidence interval; Triple-class refractory: defined as refractory to ≥1 immunomodulatory drug, ≥1 proteasome inhibitor, and ≥1 anti-CD38 monoclonal antibody. Penta-refractory:

Supplemental Fig 2. DOR according to STP.

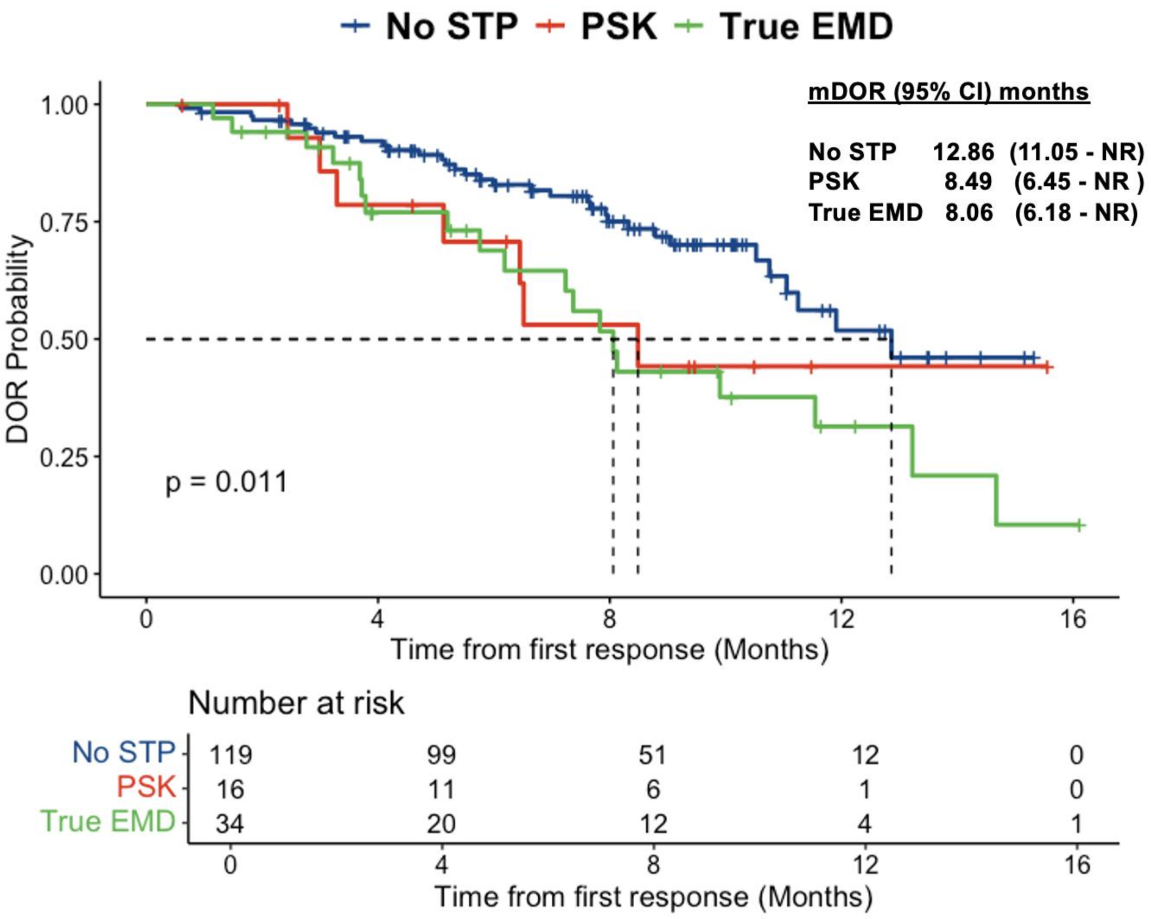

Supplement: Supplementary file 1 — Supplemental Material [file 41408_2025_1414_MOESM1_ESM.pdf]
